# Feline Immunodeficiency Virus in South America

**DOI:** 10.3390/v4030383

**Published:** 2012-03-14

**Authors:** Bruno M. Teixeira, Mitika K. Hagiwara, Juliano C. M. Cruz, Margaret J. Hosie

**Affiliations:** 1 Department of Pathology and Center for Biodefense and Emerging Infectious Diseases, The University of Texas Medical Branch, 301 University Boulevard, Galveston, TX 77555-0609, USA; 2 Department of Medical Clinics, College of Veterinary Medicine, University of São Paulo, Av. Prof. Dr. Orlando Marques de Paiva 87, 05508-270 São Paulo, SP, Brazil; Email: mkhagiwara@usp.br; 3 Retrovirus et Pathologie Comparee, Universite Lyon 1, 50 Avenue Tony Garnier, 69007 Lyon, France; Email: jcminardi@yahoo.com; 4 Retrovirus Research Laboratory, Institute of Comparative Medicine, Faculty of Veterinary Medicine, University of Glasgow, Henry Wellcome Building for Comparative Medical Sciences, 464 Bearsden Road, Glasgow G61 1QH, UK; Email: margaret.hosie@glasgow.ac.uk

**Keywords:** feline immunodeficiency virus, South America, nondomestic felids, domestic cats

## Abstract

The rapid emergence of AIDS in humans during the period between 1980 and 2000 has led to extensive efforts to understand more fully similar etiologic agents of chronic and progressive acquired immunodeficiency disease in several mammalian species. Lentiviruses that have gene sequence homology with human immunodeficiency virus (HIV) have been found in different species (including sheep, goats, horses, cattle, cats, and several Old World monkey species). Lentiviruses, comprising a genus of the *Retroviridae* family, cause persistent infection that can lead to varying degrees of morbidity and mortality depending on the virus and the host species involved. Feline immunodeficiency virus (FIV) causes an immune system disease in domestic cats (*Felis catus*) involving depletion of the CD4+ population of T lymphocytes, increased susceptibility to opportunistic infections, and sometimes death. Viruses related to domestic cat FIV occur also in a variety of nondomestic felids. This is a brief overview of the current state of knowledge of this large and ancient group of viruses (FIVs) in South America.

## 1. Introduction

Feline immunodeficiency virus (FIV) is a *Lentivirus*, closely related to HIV and SIV, which infects members of *Felidae* family. FIV is an important viral pathogen worldwide in the domestic cat (*Felis catus*), causes a slow progressive degeneration of immune functions that eventually leads to a disease. FIV is unique among the nonprimate lentiviruses because in its natural host species it induces a disease similar to AIDS in humans infected with human immunodeficiency virus type 1 (HIV-1), characterized by a progressive depletion of CD4^+^ T lymphocytes [5,28,35,48,77]. Species-specific strains, related to domestic cat FIV, have been isolated from a variety of nondomestic *Felidae* [11,43]. Like HIV, FIV can be transmitted via mucosal exposure, blood transfer, and vertically either prenatally or postnatally [26]. For these reasons, FIV has been studied widely as both an important veterinary pathogen and an animal model for HIV/AIDS. 

**Figure 1:**
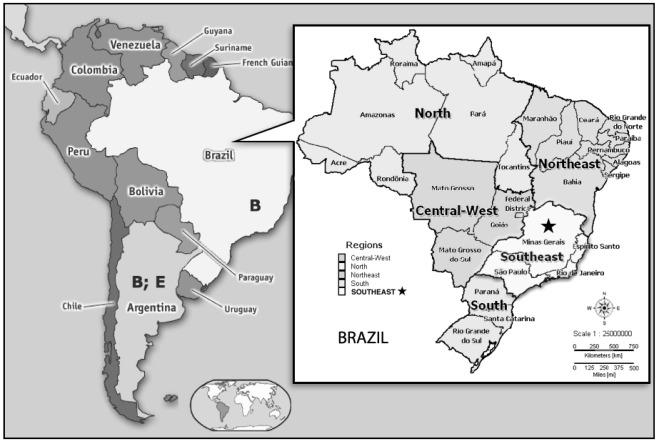
Distribution of domestic cat immunodeficiency virus (FIV) subtypes in South America. The area where all phylogenetic studies were carried out in Brazil is highlighted (★).

Although FIV was first recognized in 1993 in Brazil [23] and in 1994 in Argentina [65], there are few data describing the prevalence, ecology, clinical aspects, or genetic analyses of FIV in South America (Figure 1). The prevalence of FIV within the continent is summarized in Table 1. A better characterization of FIV strains circulating within South America will be required to augment our understanding of the importance of this lentivirus in felids. This paper provides an overview of the current state of knowledge of this large and ancient group of viruses (FIVs) in South America, grouped according to domestic and nondomestic felids. The data obtained allow a better understanding on FIV epidemiology and distribution. Efforts were made to gather and review all of the available information for each country.

**Table 1:** Epidemiologic studies of Feline immunodeficiency virus (FIV) from South America.

Reference	Year	Country / Geographical distribution	Technique	Felid species	N^o ^tested	% Positive
(43)	1992	Brazil/Chile	Western Blot	*Puma concolor*	18	0
(65)	1994	Argentina	Western Blot	*Felis catus*	26	34.6
(52)	1997	Brazil—São Paulo	ELISA	*Felis catus*	401	11.7
(9)	2000	Brazil—Rio Grande do Sul	PCR	*Felis catus*	40	37.5
(61)	2002	Brazil—Rio de Janeiro	ELISA	*Felis catus*	126	16.6
(18)	2003	Brazil—São Paulo	ELISA	*Leopardus pardalis, L. tigrinus, L. wiedii, Herpaiturus yaguarondi, Oncifelis geoffroyi*	104	0
(12)	2003	Brazil—Minas Gerais	PCR	*Felis catus*	450	2.66
(64)	2007	Brazil—Minas Gerais	PCR	*Felis catus*	145	4.14
(19)	2006	Brazil—Roraima; Acre; Mato Grosso; Mato Grosso do Sul; São Paulo; Rio de Janeiro	ELISA/Western Blot	*Puma concolor; Leopardus pardalis; Leopardus tigrinus*	21	4.76/9.52
(21)	2007	Bolivia—Chaco	ELISA	*Leopardus pardalis, Oncifelis geoffroyi, Herpaiturus yaguarondi*	20	0
(31)	2008	Brazil—São Paulo	PCR	*Felis catus*	454	14.7
(36)	2008	Ecuador—Galapagos	ELISA	*Felis catus*	52	0
(20)	2011	Brazil—São Paulo	ELISA/Western Blot	Different species of neotropic and exotic felids	145	6.9 *

*9 lions and 1 Geoffroy’s cat.

## 2. Felis Catus

**Figure 2:**
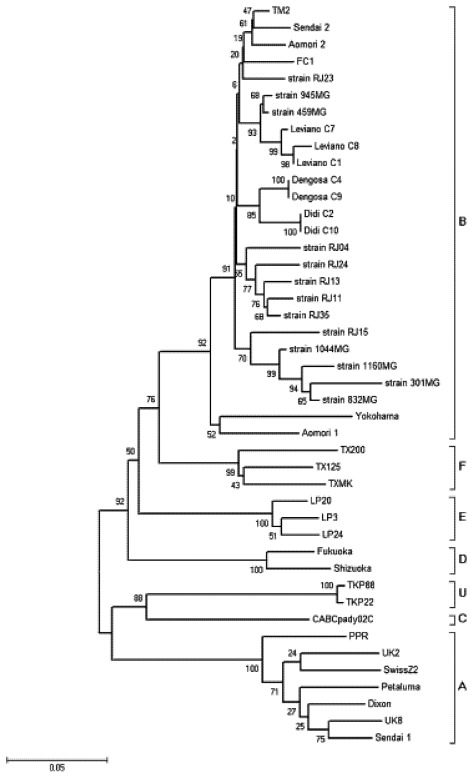
Phylogenetic tree of 473 –bp sequences from the V3-V4 region of FIV-Fca *env*. The subtype of the obtained sequences was determined by phylogenetic analysis, using an unrooted neighbor joining tree with Kimura2-parameter genetic distances and bootstrap analysis with 1000 iterations to evaluate clad consistency.

FIV infection, in domestic cats, causes a variable immunodeficiency syndrome characterized by recurrent gingivitis-stomatitis, cachexia, wasting, neurology, and an increased incidence of tumor development [1,4,48,76]. In contrast, the ungulate lentiviruses cause diseases reminiscent of chronic inflammatory conditions while infection with the bovine lentivirus seems to be inapparent [71]. The rate of progression of the disease can depend on the genotype of the infecting FIV and is also likely influenced by undefined genetic determinants of the particular host [16]. FIV infection in domestic cats is associated with early robust humoral and cellular anti-viral immune responses, followed by a progressive immune suppression that results eventually in AIDS. The outcome of infection depends on the balance between the viral destruction of the immune system and the ability of the remaining immune system to eliminate the virus. Although the decrease in numbers of CD4+ cells is the hallmark of FIV infection, the virus has been shown to infect a variety of cell types in their respective hosts including CD4^+^ and CD8^+^ lymphocytes, B lymphocytes, cells of neuronal lineage and monocyte/macrophage lineage [15,17,29]. Joshi *et al.* (2005) have characterized feline CD4^+^ CD25^+^ T regulatory cells that support FIV replication. Recently, Reggeti, Ackerley and Bienzle (2008) have shown that feline dendritic cells express specific viral receptors and are infected productively by FIV [53]. FIV shares a similar pattern of receptor usage to HIV-1; however, CD 134 rather than CD4 is the primary binding partner, and subsequent interaction with the secondary receptor CXCR4 permits cells entry [58,72,73]. Differences in pathogenicity have been demonstrated among genetically distinct subtypes of FIV that circulate in domestic cats [14,16,49,63,68,73]. On the basis of the analysis of envelope glycoprotein (Env), focusing on the third to fifth variable regions (V3-V5), FIV has been classified into five subtypes [30,46,60] a number that should be expected to increase as further studies reveal additional diversity. Recent studies identified distinct groups of FIV isolates from the United States and New Zealand [24,69] (Figure 2).

Data regarding FIV infection in domestic felids in South America are sparse and have not been well evaluated. Expanded surveys of South American isolates will be required to determine the FIV isolates in the continent since only few studies have been published. Although there are no doubts about the presence of FIV in South America, prevalence data obtained using different techniques cannot be compared amongst countries or studies (Table 1). Knowing the prevalence and variability of FIV is important for designing and testing vaccines under field conditions [27,77]. Also, identification of circulating subtypes is essential to develop strategies for molecular diagnosis, since the genetic diversity of this virus is high [44,54] which may lead to false negative diagnoses if inappropriate primers are used. In South America, only subtype B and E viruses have been found. It is important to remember that subtype B viruses are distributed worldwide and that the subtype E viruses have been more consistently identified only in Argentina (Figure 1).

Preliminary studies suggested that FIV infection is widespread in the domestic cat population of Brazil [9,12,40,52,61,64]. A published review indicated that subtype E was the only prevalent in Brazil [77]. Nevertheless, all studies indentified B as the only subtype circulating in FIV positive animals in Brazil, [12,32,40,62]. Here an analysis was conducted with 473 bp of sequence encoding 157 amino acids comprising the V3-V4 region of the envelope glycoprotein from different subtypes, including those reported previously from South America (Figure 2). In this study we used this region of *env* in order to permit us to include more samples from South America. For this phylogenetic tree, the GenBank accession numbers, names, country and subtype for the FIV *env* sequences included were: M25381.1, Petaluma, United States, A; L00608.1, Dixon, United States, A; M59418.1, TM2, Japan, B; M36968.1, PPR, United States, A; X69496.1, UK8, England, A; X69494, UK2, Scotland, A; X57001, SwissZ2, Switzerland, A; AY621093, FC1, United States (Florida), B; U02392.1, CABCpady02C, Canada, C; D84498, LP20, Argentina, E; D84496, LP3, Argentina, E; D84500, LP24, Argentina, E; D37813.1, Sendai 1, Japan, A; D37816, Aomori 1, Japan, B; D37814.1, Sendai 2, Japan, B; D37812, Yokohama, Japan, B; D37815.1, Fukuoka, Japan, D; D37817.1, Aomori 2, Japan, B; D37811.1, Shizuoka, Japan, D; AY139094.1, TX125, United States (Texas), F; AY139096.1, TX200, United States (Texas), F; AY139097.1, TXMK, United States (Texas), F; EF153977.1, TKP88, New Zealand, U; EF153979.1, TKP22, New Zealand, U; EU375619, RJ35, Brazil, B; EU375617, RJ24, Brazil, B; EU375616, RJ23, Brazil, B; B; EU375597.1, strain RJ04, Brazil, B; EU375604.1, strain RJ11, Brazil, B; EU375606.1, strain RJ13, Brazil, B; EU375608.1, strain RJ15, Brazil, B; DQ248885.1, strain 1044MG, Brazil, B; DQ177159.2, strain 945MG, Brazil, B; DQ641681.1, strain 459MG, Brazil, B; DQ865447.1, strain 301MG, Brazil, B; DQ865449.1, strain 832MG, Brazil, B; DQ865454.1, strain 1160MG, Brazil, B.

It is important to state that all phylogenetic studies carried out in Brazil were performed in the same area, namely the south-east, and that Brazil is a huge country (Figure 1). More widespread surveys of Brazilian isolates are required to determine whether a single subtype of FIV predominates in Brazil. In Brazilian domestic cats, FIV infected cats have been observed over a prolonged period. During this time, few clinical signs were observed, although the virus was replicating and inducing changes in the immune system, leading to a progressive decline in immune function and the development later of overt clinical signs [51, 78, Hagiwara and Teixeira, unpublished data]. Previously, Brazilian studies established relationships between FIV infection and *Toxoplasma gondii* and *Mycoplasma haemofelis* [37,39]. Otherwise, no association with disease has been recorded in cases of Brazilian FIV infection. It has been suggested that clade B viruses may be more ancient and relatively host adapted and thus may be less virulent [2,50,63].

Preliminary seroepidemiological studies carried out on clinical cases suggested that FIV infection is widespread in the domestic cat population of Argentina [65]. The genetic diversity of FIV isolates from Argentine domestic cats has been well characterized [47,75]. FIV isolates were isolated from peripheral blood mononuclear cells of four domestic cats. Phylogenetic analysis revealed that one isolate clustered with subtype B and the others formed subtype E [47], prototype sequence for this group (Figure 2).

In the north of the continent a single study was performed in 52 domestic cats on Isabela Island, Galapagos, Ecuador’s coast. It was demonstrated using serological methods that none of the tested animals was infected with FIV [36].

## 3. Nondomestic Felid Species

Viruses related to domestic cat FIV occur also in nondomestic felids, indeed FIV strains have been present in the nondomestic cat population for longer than domestic cats [45]. Carpenter *et al.* (1996) comment that members of at least eighteen of the 37 species in the family *Felidae* carry an FIV-related virus, as has been shown by the presence in their sera of antibodies which react with FIV antigens. A further twelve species were reported in another study that employed a three-antigen Western blot screening (cat, puma and lion FIV antigens) and a multigene PCR amplification of FIV genes [66]. In South America, 12 native species of Felidae’s family are found: *Leopardus braccatus*; *Leopardus colocolo*; *Leopardus geoffroyi*; *Leopardus guingna*; *Leopardus jacobita*; *Leopardus pajeros*; *Leopardus pardalis*; *Leopardus tigrinus*; *Leoparuds wiedii*; *Puma concolor*; *Puma yagouaroundi*; *Panthera onca* [74]. Lentiviruses in eight of these species have been detected in South America [6,10,19,20,33,55,66].

Data regarding FIV infections in South American wild felids are sparse and studies have concentrated primarily on Brazil. The presence of antibodies against FIV in puma, detected by Western blotting, was found in Argentina (5 in 22, 23%), Bolivia (5 in 5, 100%), Brazil (2 in 13, 15%), Peru (1 in 5, 20%) and Venezuela (4 in 8, 50%) [10]. Further studies have reported antibodies recognizing FIV and the puma lentivirus (PLV in Brazilian free-ranging puma) [6,19]. Troyer *et al.* (2005) concluded that most of the South American felids maintain a low level of FIV infection throughout their population. Within wild populations, the seroprevalence in South American felids varies from 5 to 28%. Unfortunately, the authors did not describe the regions of the continent where the samples originated. FIV *pol* genes from a Peruvian and a Brazilian zoo puma have been sequenced, the former being classified as subtype B and the latter as a distinct group, neither A nor B [10]. Additionally, FIV provirus has been reported in Brazilian jaguars (*Panthera onca*), pumas, jaguarondis (*Puma yagouaroundi*), oncelots (*Leopardus pardalis*), margays (*Leopardus wiedii*), pampas cat (*Leopardus colocolo*), geoffroy’s cat (*Leopardus geoffroyi*) and little spotted cats (*Leopardus tigrinus*) [20,33,55]. The finding of these FIV infected species highlights the need for additional monitoring. Although the implications of these infections for wild felid conservation are difficult to assess, it is generally accepted that monitoring these infections is an important component for the management of endangered populations [13].

It is important to emphasize that FIV strains infecting 9 species of the *Felidae* have been at least partially sequenced and molecularly characterized [3,10,11,25,34,38,42,43,66]. Genetic analysis indicates that different felid species are infected by different strains of FIV [8,11]. Analysis of *pol* gene sequence of FIV from lions (*Panthera leo*), pumas (*Puma concolor*) and domestic cats indicated that each species has a specific strain of FIV and that the strains are related but distinct [7,43]. Also, strains from African lions (subtype B and E) differ in their abilities to replicate in feline cell lines [59], their sensitivity to receptor antagonists [71], and their requirement for ectopic expression of CD134, the primary cellular receptor, for productive infection [41].

It remains to be demonstrated that FIV-related viruses cause severe disease in species other than the domestic cat [6,38]. The apparent absence of clinical signs in pumas and lions may reflect a longer period of coevolution between virus and host in these species, whereas in the domestic cat, the virus and host have not yet had time to reach a similar state of nonpathogenic coexistence [6,7,57]. However, it is by no means certain that FIV does not cause disease in non-domestic cats. Not long ago, reports have shown immune depletion associated with FIV infection in lions and pumas [56,57] and another recent study reported evidence of immune suppression in the Pallas’ cat (*Otocolobus manul*), including histopatological changes [8]. In addition, interspecies transmission (although is rare) may occur [22,67]. For example, a leopard cat (*Felis bengalensis*) was found to be infected with a domestic cat virus [42] and FIV infecting one puma was more characteristic of domestic cat FIV rather than puma FIV [10].

## 4. Conclusions

The prevalence of FIV infection is South America has not been well evaluated and regional variations remain largely unexplored in domestic and wild cats. Considering that FIV has been detected in domestic cats in South America and that wild and domestic cats have overlapping territories in the communities and buffer zone, there is the potential for domestic felids to transmit this virus to naive wild felids in zoologic as well as free-range settings. The isolation and molecular characterization of these pathogens, both in domestic and a variety of wild felines, would be helpful and may provide important baseline data to develop effective programs aimed at infectious disease prevention. We believe that the feline population should be continually monitored for FIV infection and that clinical correlates to FIV infection should be further investigated. As recently proposed [70], researchers could consider early surveillance programs across defined populations and detailed, cohort studies of naturally infected animals to provide further insights. Such studies would provide an opportunity to track retrospectively the pattern and consequences of an ongoing epizootic. There are technical reasons that hinder such studies, there is an urgent need for increased capacity in South American laboratories in order to conduct FIV screening and the apparent absence of FIV infection in some countries of the continent may merely reflect an absence of investigations. In addition, it is not easy to study FIV in wild cats as it is difficult to obtain samples from wild populations and only when these difficulties are overcome will it be possible to analyze and characterize FIV strains from the continent.
